# Substitution of physicians by nurses in primary care: a systematic review and meta-analysis

**DOI:** 10.1186/1472-6963-14-214

**Published:** 2014-05-12

**Authors:** Nahara Anani Martínez-González, Sima Djalali, Ryan Tandjung, Flore Huber-Geismann, Stefan Markun, Michel Wensing, Thomas Rosemann

**Affiliations:** 1 Institute of Primary Care, University Hospital Zurich, Pestalozzistrasse 24, 8091 Zurich, Switzerland; 2 Scientific Institute for Quality in Healthcare, Radboud University Medical Centre, P.O. Box 9101, 6500 HB Nijmegen, Netherlands

**Keywords:** Systematic review, Meta-analysis, Physician-nurse substitution, Skill-mix, Health outcomes, Cost

## Abstract

**Background:**

In many countries, substitution of physicians by nurses has become common due to the shortage of physicians and the need for high-quality, affordable care, especially for chronic and multi-morbid patients. We examined the evidence on the clinical effectiveness and care costs of physician-nurse substitution in primary care.

**Methods:**

We systematically searched OVID Medline and Embase, The Cochrane Library and CINAHL, up to August 2012; selected and critically appraised published randomised controlled trials (RCTs) that compared nurse-led care with care by primary care physicians on patient satisfaction, Quality of Life (QoL), hospital admission, mortality and costs of healthcare. We assessed the individual study risk of bias, calculated the study-specific and pooled relative risks (RR) or standardised mean differences (SMD); and performed fixed-effects meta-analyses.

**Results:**

24 RCTs (38,974 participants) and 2 economic studies met the inclusion criteria. Pooled analyses showed higher overall scores of patient satisfaction with nurse-led care (SMD 0.18, 95% CI 0.13 to 0.23), in RCTs of single contact or urgent care, short (less than 6 months) follow-up episodes and in small trials (N ≤ 200). Nurse-led care was effective at reducing the overall risk of hospital admission (RR 0.76, 95% CI 0.64 to 0.91), mortality (RR 0.89, 95% CI 0.84 to 0.96), in RCTs of on-going or non-urgent care, longer (at least 12 months) follow-up episodes and in larger (N > 200) RCTs. Higher quality RCTs (with better allocation concealment and less attrition) showed higher rates of hospital admissions and mortality with nurse-led care albeit less or not significant. The results seemed more consistent across nurse practitioners than with registered or licensed nurses. The effects of nurse-led care on QoL and costs were difficult to interpret due to heterogeneous outcome reporting, valuation of resources and the small number of studies.

**Conclusions:**

The available evidence continues to be limited by the quality of the research considered. Nurse-led care seems to have a positive effect on patient satisfaction, hospital admission and mortality. This important finding should be confirmed and the determinants of this effect should be assessed in further, larger and more methodically rigorous research.

## Background

Concerns about the global shortage of health care providers [1,2] continue to fuel the debate about the need to introduce new strategies of health care delivery. Especially, the increasing shortage of physicians makes substitution by nurses a common demand which is expected to escalate with ageing populations and an increasing prevalence of chronic conditions. Two systematic reviews published ten years ago suggested that care provided by nurses might be equally good as the care provided by physicians [3,4]. Health outcomes, use of resources and healthcare costs were found to be similar between nurses and physicians while patient satisfaction was similar or better with nurse-led care. These differences, however, were limited by the low volume and quality of the studies. In this context, it is also important to consider that nurses’ education continues to evolve resulting in different roles and qualifications across different health care systems. It seems timely therefore to assess whether the updated evidence would support the notion that nurses can substitute physicians in specific clinical tasks. Therefore, we performed a systematic review and meta-analysis of trials investigating the clinical effectiveness and costs of nurses working as substitutes for physicians in primary care.

## Methods

We followed a protocol developed prior to starting the review and followed the PRISMA guidelines [5] for the reporting of systematic reviews and meta-analyses (Additional file 1: Table S1).

### Study inclusion/exclusion criteria

We included peer reviewed randomised controlled trials (RCTs) from any country published in English in which nurses (in any type of role) substituted physicians by acting as the main figure of care with autonomous or delegated clinical responsibility for tasks that would have formerly been performed by physicians alone: where nurse-led care was compared to physician-led care (family physicians, paediatricians, and geriatricians); the intervention had taken place in general practices, community or ambulatory care settings; in patients of all ages seeking care for all conditions including mental health and addiction restricted to primary care; and which reported on patient satisfaction, quality of life (QoL), hospital admission, mortality and cost of health services. Following the framework published in a Cochrane review [3], we excluded studies in which nurses firstly, provided services which supplemented or extended the care provided by physicians or tasks that are not part of the usual care of physicians and secondly, where nurses collaborated with other clinicians in a team and thus the effect of nurse-led care, as the main intervention, could not be distinguished.

### Study identification

We searched OVID Medline, Embase, CINAHL and The Cochrane Library of Systematic Reviews which includes the Cochrane Effective Practice and Organisation of Care Group, from all available dates until August 2012. The searches, not age-, date- or country-specific included ‘primary care’, ‘skill-mix’, ‘physicians’-‘nurse’ substitution’ (Additional file 1: Table S2). We also manually searched the reference lists of included studies and relevant reviews.

### Assessment of study quality

We assessed the risk of bias of all trials without the calculation of a composite score following available guidelines [6-8]. We considered bias due to attrition of more than 20% to be of significant concern; and adequate intention-to-treat (ITT) if trial authors analysed participants based on their original group allocation regardless of protocol violations or non-compliance [9].

### Data extraction

Both qualitative (characteristics of studies, population and interventions) and numeric data (dichotomous and continuous format) were extracted using structured data collection forms, designed and pilot-tested *a-priori*. If more than one comparison group of interest were reported, these were combined and compared as one to nurse-led care. If the results from a single study were reported in more than one publication, data were extracted as one study. When one publication reported more than one cohort, data were extracted as separate studies.

### Selection and assessment of studies and acquisition of data

Two authors independently screened titles and abstracts, assessed both the full-text of eligible publications and the risk of bias of included studies, and extracted data. Differences were resolved through consensus.

### Statistical analyses

We calculated the individual and pooled unadjusted relative risks (RR) and the standardised mean differences (SMD); and performed meta-analyses when at least three trials reported appropriate data, using the inverse variance fixed-effects (FE) method and repeated the analyses using a random-effects (RE) model in Cochrane RevMan (Version 5.1) [10]. We report the summary statistics, their 95% confidence intervals (CI) and consider p < 0.05 statistically significant. When scales pointed in opposite directions, we subtracted the mean from the maximum possible value of the scale and estimated the standard deviations (SD) using well-established techniques [11]. We analysed dichotomous and continuous data together by converting ORs to an effect size expressed in SMD using available methods [12]. We decided to use a FE model in keeping with: 1) having no basis to assume that the effects had a normal distribution, 2) the small number of studies in at least two of the analyses, 3) the accuracy in estimates and CIs that FE provides even in a small number of studies and the more weight assigned to larger studies; RE gives similar weight to small and larger studies. We quantified heterogeneity using the I^2^ statistic [13] and explored the effects of nurse-led care and potential sources of I^2^ by pooling data into pre-specified subgroup analyses by clinical characteristics: nurses’ roles (based on reported details: nurse practitioner with higher degree courses/specialisation (NP+) *versus* nurse practitioner (NP) *versus* registered/licensed nurse (RN/LN), type of care (single contact *versus* on-going care; urgent *versus* non-urgent) and length of follow-up (months: <6 *versus* ≥6; <12 *versus* ≥12). We explored the effect of potential sources of bias by study size (small, N < 200 *versus* large, N ≥ 200), allocation concealment (adequate *versus* inadequate/unclear) and attrition (<20% *versus* ≥20%), and inspected publication bias using funnel plots where there were at least 10 trials [14]. We performed sensitivity analyses by excluding trials with potentially contaminated samples (i.e. patient crossover between groups), quasi and cluster design and in which nurses had full clinical autonomy (to perform tasks) and/or where this information was not reported. For data not combined in meta-analyses, individual trial estimates were compared.

## Results

### Study identification

**Figure 1:**
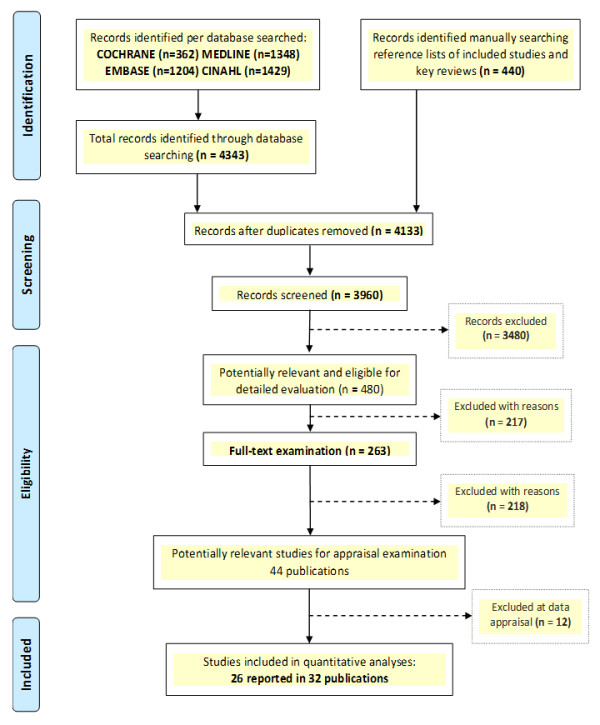
PRISMA Flow Diagram - study selection process.

A total of 4,133 original records were identified. We excluded 12 of 44 relevant publications for the reasons provided in Table S3 (Additional file 1). Twenty-six studies reported in 32 publications met the inclusion criteria and comprised a total of 38,974 randomised participants (Figure 1) [15-46]. Twenty-four of the studies were RCTs and the other two were economic evaluations based on three of the appraised RCTs [17,26,38]. Table 1 and Table S4 (Additional file 1) report the summary and detailed characteristics of participants, interventions and outcomes of the trials included in review.

**Table 1:** Summary characteristics of participants and interventions of studies included in review

**Study**			**Setting**	**Participants**	**Nurses’ group**				**Physicians’ group**				**Intervention**			**Outcomes reported**				
**Location, first author, year**	Design, period^*^	**FUP, m**	**Facilities, n**	**Included diagnosis**	**Nurses, n**	**Patients, N**	**Mean age (SD), y**	**Male, %**	**Phys., n**	**Patients, N**	**Mean age (SD), y**	**Male, %**	**Nurses’ training/experience**	**FCA**	**GDL**	**PS**	**QoL**	**HA**	**M**	**C**
ZA 2	cRCT, 2008-2010.	18	Nurse ART clinic, 31.	HIV/AIDS.	103	6415	38 (8.9)	30	nr	6479	38 (9.63)	27	Middle nurse managers trained to assume responsibility for ART and established patients’ eligibility for ART.	no	yes			✓	✓	
Fairall, 2012 [36], cohort 2																				
ZA 1	cRCT, 2008-2010.	16-18	Nurse ART clinic, 31.	HIV/AIDS.	103	6159	36 (9.6)	33	nr	4923	35 (9.63)	31	Middle nurse managers trained to assume responsibility for ART and established patients’ eligibility for ART.	no	yes			✓	✓	
Fairall, 2012 [36], cohort 1																				
NL 6	RCT, period nr.	14	Practice, 1.	Diabetes Mellitus Type II.	2	116	67.1 (11)	53	5	114	69.5 (10.6)	42	Practice nurse with one week training in diabetes mellitus; nurse had no special training in the treatment of diabetes prior to starting trial.	yes	yes	✓	✓			
Houweling, 2011 [30]																				
NL 5	RCT, 2006-2008.	24	Hospital outpatients, 1; Practice, 18.	Asthma.	nr	36	11.2 (2.9)	64	nr	71 (37^§^, 34^‡^)	11.2 (2.5) ^§^; 10.1 (2.6)^‡^	58	Asthma nurse.	no	yes		✓	✓		
Kuethe, 2011 [25]																				
RU 1	RCT, 2006 -2009.	6, 18	Medical centre practice, 1.	Heart Failure with Preserved Ejection Fracture.	10	50	66.5 (3.2)	27	8	50	68 (4.3)	34	Nurses with special degree in patient education obtained in a joint course.	no	yes		✓	✓	✓	
Andryukhin, 2010 [46]																				
NL 4	RCT, 2006-2007.	12	Healthcare centre, 6.	CVD, Hypertension, Hypercholesterolemia.	6	808	64 (9.0)	58	25	818	64 (9.0)	62	Advance practice nurse already employed to manage patients with asthma, chronic obstructive pulmonary disease, or diabetes.	nr	yes				✓	
Voogdt-Pruis, 2010 [16]																				
NL 3	RCT, 2006.	0.5	Practice, 15; Reference, 5	Common complaints.	12	817	42.8 (16.5)	38	50/17^†^	684	46.1 (16.6)	40	Nurse practitioner with Master degree in Advance Nursing trained in common complaints.	no	yes	✓	✓			✓
Dierick-Van Dale, 2009 [39]																				
UK 9	RCT, 2002-2004.	6	Nurse clinic, 1	GORD, moderate Gastritis.	nr	89	50.2 (13.9)	49	nr	86	48.4 (12.8)	49	Gastrointestinal nurse practitioner.	no	yes		✓			✓
Chan, 2009 [42]																				
NL 2	cRCT, period nr.	12	nr.	All forms of incontinence.	1	38	51 (13.0)	0	28^¶^	13	51 (13.0)	0	Registered nurse specialist in incontinence.	no	yes	✓	✓			
Du Moulin, 2007 [37]																				
US 6	RCT, period nr.	6	Community, 2; PHD, 1.	Diabetes Mellitus.	nr	95	55.7 (13.1)	32	108^#^	102	57 (11.4)	35	Diabetes nurse.	no	yes				✓	
Hiss, 2007 [32]																				
NL 1	RCT, 2000-2001.	24	Practice, 12	Asthma and COPD.	2	139	49.9 (14.2)	35	14	137	44.7 (13.6)	28	GP assistant with pre- and during-trial training to deal with the differences between asthma and COPD.	no	yes		✓			
Hesselink, 2004 [33]																				
UK 8	RCT, 2000-2001.	6	Nurse clinic hospital based.	Diabetes Mellitus Type II pre-diagnosed with Hypertension or in receipt of BPLT.	nr	60	58.1 (13.8)	57	nr	60	62.4 (9.1)	70	Hypertension nurse.	no	yes				✓	
Denver, 2003 [40]																				
UK 7	RCT, 1996-1999.	24	Practice, 438.	Parkinson's Disease.	9	1041	nr	57	nr	818	nr	56	Community nurse with a course in Parkinson Disease.	no	nr		✓		✓	✓
Jarman, 2002 [29]																				
UK 6	RCT, period nr.	4	Health Centre Practice, 1	Asthma	nr	55	Median (IQR): 35 (29-47)	56	9	46	Median (IQR): 37 (27-50)	33	Nurse with structured training in Asthma care.	no	yes		✓			
Kernick, 2002 [27]																				
US 5	RCT, 1995-1997.	6-12, 24	Community clinic, 4; Primary care clinic, 1.	Asthma, Diabetes Mellitus, Hypertension, or urgent visits.	7	1181	44	24	11	800	44.9	22	Community nurse practitioner.	no	nr	✓	✓	✓		
Mundinger, 2000 [22,24]																				
UK 5 Kernick, 2000 [28]	RCT, period nr.	4	Health Centre, 1	Psoriasis and Eczema.	1	55	47.4 (18.4)	39	nr	54	51.7 (15.8)	48	Practice nurse with training in psoriasis and eczema management.	no	yes		✓			
UK 4	RCT, period nr	0.5-1	Practice, 10	Diverse complaints.	12	1465^║^	range: 0 to >75	39	10	1465^║^	range: 0->75	42	Nurse practitioner with diploma on care for same day consultations for primary care.	no	nr	✓				
Kinnersley, 2000 [26]																				
UK 3	RCT, period nr.	0.5	Practice, 20	Diverse complaints: e.g. minor injuries, respiratory complaints.	20 (1 per practice)	651	nr	42	nr	665	nr	43	Nurse with course at BSc or MSc level.	no	nr	✓	✓			✓
Venning, 2000 [17]																				
UK 2	RCT, 1998-1999.	0.5	Practice, 5	Acute minor illnesses.	5	900	median (IQR): 26 (9-41.8)	40	19	915	median (IQR): 29.1 (9.7-44.9)	40	Practice nurse with a course in minor illnesses and piloted before study.	no	nr	✓				
Shum, 2000 [18]																				
US 4	qRCT, 1999-2001.	12	Primary care veterans affair clinic, 1	Undifferentiated conditions.	9	150	62	99	45	300	61	98	Nurse practitioner who was on staff for at least six months in primary care.	yes	yes			✓	✓	
Hemani, 1999 [34]																				
UK 1	RCT, 1995-1996.	12, 24, 56.4, 122.4	Practice, 19	Coronary Heart Disease secondary prevention.	28	673	66.1 (8.2)	58	nr	670	66.3 (8.2)	58	District and practice nurses trained in clinic protocols/GDLs for behavioural techniques change.	no	yes		✓	✓	✓	✓
Campbell, 1998 [19-21,41,43-45]																				
US 3	RCT, 1980.	0.5	Community clinic, 1	Family planning, venereal diseases, acute non-traumatic minor illnesses.	5	25	nr	nr	5	25	nr	nr	Registered professional nurse with preparation and skills in physical diagnosis, psychosocial assessment, and health-illness management in primary care.	nr	yes	✓				
Winter, 1981 [15]																				
US 2	RCT, 1971.	≥6	HO clinic, 1; Private, 3	Undifferentiated.	4	40	nr	nr	nr	20	nr	nr	Nurse clinicians with training in service delivery.	yes	nr			✓		
Flynn, 1974 [35]																				
US 1	RCT, period nr.	12	University Hospital clinic, 1; Nurse clinic, 1	Hypertension, CVD, Obesity, Arthritis, Somatization.	nr	33	range: 16-78	12	nr	33	range: 16-83	12	Nurses who provided primary source care for at least one year before the study.	no	yes	✓	✓	✓		✓
Lewis, 1967 [23]																				

Legend.

Studies are listed by year (y) of publication, in decreasing order; and labelled after the country where they were conducted.

US, United States; NL, The Netherlands; UK, United Kingdom; ZA, South Africa; RU, Russia; RCT, Randomised Controlled Trial; cRCT, cluster Randomised Controlled Trial; qRCT, quasi-Randomised Controlled Trial; FUP, follow-up episodes are reported in months (m); nr, not reported; ART, Antiretroviral Therapy; PHD, public health department; HO, Hospital Outpatients; HIV, Human Immunodeficiency Virus; CVD, Cardiovascular Disease; GORD, Gastro-Oesophageal Reflux Disease; COPD, Chronic Obstructive Pulmonary Disease; BPLT, Blood Pressure Lowering Treatment; SD, standard deviation; IQR, Interquartile Ranges;

FCA, full clinical autonomy; GDL, interventions based on clinical guidelines or protocols; PS, patient satisfaction; QoL, quality of life; HA, hospital admissions; M, mortality; C, costs.

^*^Start and end year when studies were conducted.

^†^Reference practices for comparison on economic/cost data.

^‡^Paediatricians.

^§^General physicians.

^║^Number of randomized patients per group not reported.

^¶^Nine were physicians and nineteen were supervisors.

^#^Sixty-three were for the control group.

### Study and population characteristics

There were twenty RCTs of parallel design, three cluster-RCTs, one quasi-RCT and two studies [31,38] with cost data from three of the included RCTs [17,26,39]. The trials were conducted in the UK (n = 9), the Netherlands (n = 6), the USA (n = 6), Russia (n = 1) and South Africa (n = 2). Median follow-up was 14.8 (range: 0.5 to 122.4) months with at least 12 months in fourteen trials, less than 6 months in seven and 6 to 12 months in the other three. The median number of participants was 1,624 (range: 50 to 12,894) with less than 200 in eleven trials and more than 200 in the other thirteen. Mean age was reported in twenty trials and ranged from 10 to 83 years. Twenty-two trials reported on gender and 38.3% of the participants were male; one included women only.

### Settings and interventions

A summary of settings, interventions and nurses’ roles are reported in Table 1 and Table S5 (Additional file 1). Nurses worked as physician substitutes in a range of care settings. The interventions were carried-out in general practices [17,18,26,29,30,33,39,44], nurse clinics [23,36,40,42] and in hospital-based, health care centres, specialised practices, community or university clinics [15,16,22,25,27,28,32,34,35,37,46]. In the controlled intervention, nurses were the main figure of care with autonomous or delegated responsibility in various clinical domains including a whole range of possible (undifferentiated/minor acute/common) or specific conditions (e.g. hypertension, heart failure, diabetes, HIV, etc.). In one trial, the clinical domain was assumed to represent undifferentiated care [35]. Nurses’ specific qualifications and training were not reported in sufficient detail but using the information provided by study authors we grouped nurses’ roles. Nurses’ roles were described in some detail in sixteen trials (two reported in one publication) [15-18,22,23,27-29,32,36,37,39,42,46]. Seven trials employed NP+ only [16,25,28,32,39,40,42], six employed NPs [18,22,26,30,34,44], eight employed RN and/or LN [15,23,27,29,33,35-37], one employed NP and NP+ [17], and one employed NP and LN [46]. Nurses’ interventions were guideline- or protocol-based in eighteen trials, while six had no report of having followed specific guidelines. Nurses’ clinical autonomy was obtainable from twenty-two trials. In three, nurses had full clinical autonomy to manage patients with diabetes type II [30] or undifferentiated conditions [34,35]. In the other nineteen, nurses made independent decisions to perform several tasks (e.g. adopting, initiating and prescribing treatment, ordering tests or referrals) but they still required minor support or contact with the physicians (e.g. to sign prescriptions, referrals and tests, to discuss patients’ records or to develop action plans). Although the interventions in the control group were not clearly described in at least a few trials, these were assumed to represent physicians-usual-led care. Ten trials addressed single contact care [15,39], single contact and on-going care [27,35,37], single contact and urgent care [17,18,26,34] and single, on-going and urgent care [22]. The other fourteen included patients in on-going care for complex conditions (e.g. HIV, Asthma, hypertension, heart failure, etc.).

### Risk of bias in the methods of the included studies

The overall quality of the studies varied substantially when assessed against current reporting standards [6,11] (Table 2). Only 54.3% of the trials measured the success of the intervention by defining a primary outcome. Random sequence generation was adequate in 54.0%, allocation concealment in 42.0%, blinding of patients and providers in 4% and blinding of outcome assessors in 21.0%. Patient or clinician crossover between groups was reported in 12.5% of the trials. At baseline, groups were comparable for all tested factors in 70.8% of the trials. Both inclusion and exclusion criteria were reported in 70.8%. Sample size calculation based on power (80.0% to 90.0%) was performed in 70.8%, but only ten trials held the least target sample size to achieve power in at least one outcome. Rates of missing data varied widely (range: 5.0% to 65.5%). While three did not report any attrition, more than half (13/24) of the trials had an attrition rate of at least 20%: nine had more than 20% in both arms in at least one outcome (range: 10.0 to 65.5%), three had at least 45.0% per arm, and four had more than 20% (range: 8.0 to 30.0%) in one arm with a differential rate of 9.0% to 15.0% across the treatment and control groups. Only 29.2% of the trials reported the use of intention to treat (ITT) techniques (type not always reported) to deal with missing data.

**Table 2:** Quality of methods in the studies included in review

**Study details (country, design, funding)**	**Inclusion & exclusion criteria**	**Outcome**		**Sequence generation**	**Allocation concealment**	**Blinding**	**Sample size**	**Attrition %**	**Funding**
		**1ry**	**2ry**						
ZA 2	✓	✓	✓	A	A	NP^‡^	✓^║^	≥20^#^	G
Fairall, 2012 [36] (Cohort 2)									
ZA 1	✓^†^	✓	✓	A	A	NP^‡^	✓^║^	≥20^#^	G
Fairall, 2012 [36] (Cohort 1)									
NL 6	✓^†^	✓	✓	I	A	NP	✓	<20	G
Houweling, 2011 [30]									
NL 5	✓^†^	✓		A	A	NP	✓^¶^	<20	NR
Kuethe, 2011 [25]									
RU 1	✓			U	I	^‡^	✓^¶^	≥20	None
Andryukhin, 2010 [46]									
NL 4	✓	✓		A	U	I^‡,§^	✓	<20	P/Ind.
Voogdt-Pruis, 2010 [16]									
NL 3	✓^†^			A	A	NP	NP	≥20	G
Dierick-Van Dale, 2009 [39]									
UK 9	✓^†^			A	A	NP^§^	✓	<20^#^	NR
Chan, 2009 [42]									
NL 2	✓^†^	✓	✓	U	U	NP	✓^║^	≥20	NR
Du Moulin, 2007 [37]									
USA 6	^*^			U	U	NP	NP	<20	G
Hiss, 2007 [32]									
NL 1	^*^	✓	✓	U	U	NP^§^	✓^║^	≥20	NR
Hesselink, 2004 [33]									
UK 8	^*^	✓	✓	I	I	NP	✓^¶^	<20^#^	NR
Denver, 2003 [40]									
UK 7	✓	✓	✓	A	A	NP	✓	<20	P/Ind.
Jarman, 2002 [29]									
UK 6	^*†^	✓	✓	A	U	U	✓	≥20^#^	NR
Kernick, 2002 [27]									
US 5	^*^			U	U	NP	✓^¶^	≥20	G
Mundinger, 2000 [22,24]									
UK 5	✓	✓		A	U	U	✓^¶^	≥20	Ind.
Kernick, 2000 [28]									
UK 4	✓	✓	✓	A	A	NP	✓^║,¶^	≥20	G
Kinnersley, 2000 [26]									
UK 3	✓			A	A	NP	NR^║,¶^	≥20	P
Venning, 2000 [17]									
UK 2	✓			A	A	NP	✓^¶^	≥20	G
Shum, 2000 [18]									
US 4	✓			I	I	NP	NP	U	NR
Hemani, 1999 [34]									
UK 1	✓			A	I	NP^§^	✓	≥20^#^	G
Campbell, 1998 [19-21,41,43-45]									
US 3	✓	✓		U	U	A^‡^	NR	U	NR
Winter, 1981 [15]									
US 2	^*^			U	U	NP	NR	<20	NR
Flynn, 1974 [35]									
US 1	^*^			U	U	NP^§^	NR	U^#^	G
Lewis, 1967 [23]									

Legend.

Studies are listed by year (y) of publication, in decreasing order. Blinding: whether patients, care providers and outcome assessors were blinded. *Attrition* of more than 20% is of significant concern. *Intention to treat* (ITT) whether study authors analysed all patients based on their original group allocation regardless of protocol violations or non-compliance. US, United States; NL, The Netherlands; UK, United Kingdom; ZA, South Africa; RU, Russia; I, Inadequate; A: Adequate; U, Unclear; NP, Not Performed; NR, Not reported; Funding, Government (G), Industry (Ind.) or Private (P) grant.

^*^Only the inclusion criteria was reported.

^†^Not all factors tested at baseline were comparable between groups.

^‡^Fairall et al. (2012) [36] partly blinded data analysts; Andryukhin et al. (2010) [46] blinded clinicians not patients; Voogdt-Pruis et al. (2010) [16] blinded patients not clinicians; Winter (1981) [15] blinded patients and clinicians.

^§^Outcome assessors blinded for some or all outcomes.

^║^Used a cluster effect approach (e.g. Huber-White).

^¶^Reached the least target sample required to achieve power.

^#^Used ITT strategies to deal with missing data.

### Effectiveness of interventions

#### Patient satisfaction with quality of care

**Figure 2:**
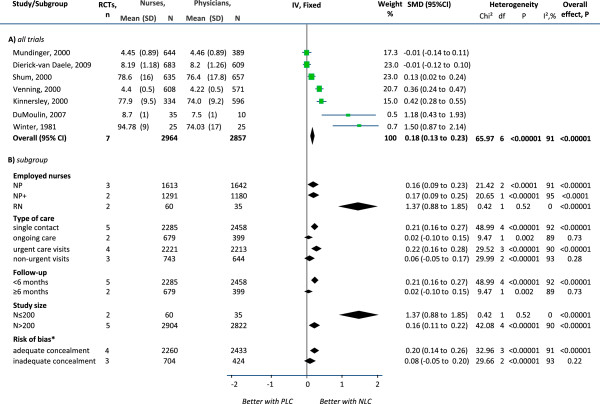
**Effects of physician-nurse substitution on patient satisfaction in A) all trials and by B) subgroups. **Legend. CI, confidence interval; df, degrees of freedom; N, total number of patients; SMD, standard mean differences; SD, standard deviation; Chi^2^, statistical test for heterogeneity; P, p-value of Chi^2^ (evidence of heterogeneity of intervention effects); I^2^, amount of heterogeneity between trials; Overall P, p-value for significance of effects of interventions; NLC, Nurse-Led Care; PLC, Physician-Led Care; NP, Nurse Practitioner; NP+, Nurse Practitioner with higher degree/courses/specialisation; RN, Registered Nurse. ^*^All trials had ≥20% attrition in at least one arm.

Patient satisfaction questionnaires were either validated [18,22,26,30], developed for the study purpose [15] or had unclear validation [17,23,37,39]. Meta-analysis of seven studies showed a significant increase in the mean satisfaction scores with nurse-led care (SMD 0.18, 95% CI 0.13 to 0.23) and significant heterogeneity between trials (I^2^ = 91%; χ^2^_6df_ = 65.97; p < 0.00001) (Figure 2). Subgroup analyses by clinical characteristics showed that RN had a stronger effect than NPs in increasing patient satisfaction, although the pooled CIs became wider due to both the smaller number of studies and smaller sample sizes (SMD 1.37, 95% CI 0.88 to 1.85). The effect estimate also increased in studies of single contact care, urgent care visits and shorter (less than 6 months) follow-up episodes, but the significance of the findings did not change. On the other hand, the effect disappeared in studies of on-going care, non-urgent care visits and longer (greater than 6 months) follow-up episodes. Subgroup analyses by study quality showed a more modest estimate with the same level of significance in larger trials, which are less prone to small study bias (N ≥ 200: SMD 0.16, 95% CI 0.11 to 0.22; N < 200: SMD 1.37, 95% CI 0.88 to 1.85). The effect was not significant in trials with inadequate allocation concealment. All trials had at least 20% attrition. Heterogeneity disappeared in the subgroup of registered nurses and smaller trials (N < 200). Two other trials with qualitative data reported significantly higher patient satisfaction scores with nurse-led care [23,30].

#### Hospital admissions

**Figure 3:**
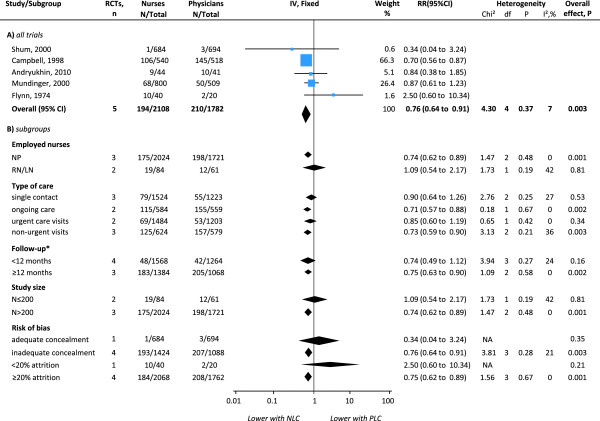
**Effects of physician-nurse substitution on hospital admissions in A) all trials and by B) subgroups. **Legend. CI, confidence interval; df, degrees of freedom; N, number of patients with events; Total, total number of patients per group; RR, Relative Risk; Chi^2^, statistical test for heterogeneity; P, p-value of Chi^2^ (evidence of heterogeneity of intervention effects); I^2^, amount of heterogeneity between trials; Overall P, p-value for significance of effects of interventions; NLC, Nurse-Led Care; PLC, Physician-Led Care; NP, Nurse Practitioner; NP+, Nurse Practitioner with higher degree/courses/specialisation; RN, Registered Nurse. ^*^Two RCTs provided data for different follow-up episodes and were incorporated accordingly: Andryukhin et al. (2010) [46] reported data at 6 and 18 months and Mundinger et al. (2000) [22,24] reported data at 6 and 12 months.

Five trials had sufficient data for meta-analysis (Figure 3), two of which reported different follow-up episodes [22,46]. The pooled RR showed a significant reduction in the risk of all-cause hospital admissions with nurse-led care (RRs 0.76, 95% CI 0.64 to 0.91) and no significant heterogeneity between trials (I^2^ = 7%; χ^2^_3df_ = 4.30; p = 0.37). Subgroup analyses by clinical characteristics showed that NPs had a positive effect in reducing all-cause admissions to hospital (RRs 0.74, 95% CI 0.62 to 0.89) while the effect was not significant with RNs. The estimate increased in studies of on-going care, non-urgent visits and longer (at least 12 months) follow-up episodes. The effect disappeared in trials of single contact care, urgent care and shorter (less than 12 months) follow-up episodes. Subgroup analyses by study quality showed that in large trials (less prone to bias) nurse-led care had an increasingly significant effect in reducing hospital admissions (N < 200: RR 1.09, 95% CI 0.54 to 2.17; N ≥ 200: RR 0.74, 95% CI 0.62 to 0.89). However, trials that were of higher quality in other ways (e.g. better allocation concealment and less attrition) tend to show the opposite effect with better quality being associated with higher rates of admissions with nurse-led care, albeit non-significant. Heterogeneity remained non-significant across subgroups and disappeared in studies of nurse practitioners, on-going and urgent care, longer follow-up episodes, larger trials and trials with at least 20% attrition. In addition, data that were not pooled showed less hospital admissions with nurse-led care [23,36] or no significant differences between groups [22,44,36] (see Additional file 1: Table S7). Qualitative data reported less hospital admissions with nurse-led care at 24 months [25] or no significant differences between groups at 1 month [26] or 12 months [34].

#### Mortality

**Figure 4:**
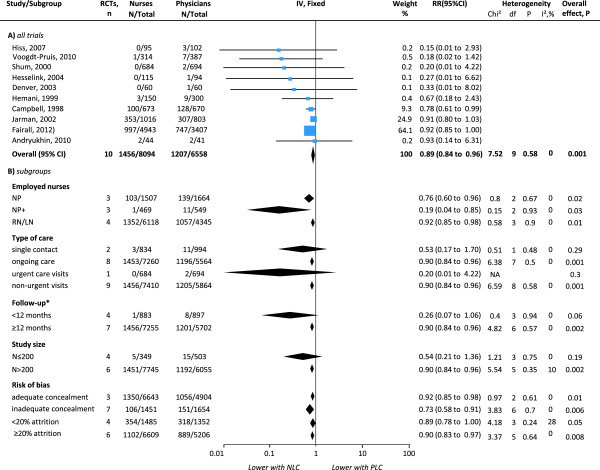
**Effects of physician-nurse substitution on mortality in A) all trials and by B) subgroups. **Legend. CI, confidence interval; df, degrees of freedom; N, number of patients with events; Total, total number of patients per group; RR, Relative Risk; Chi^2^, statistical test for heterogeneity; P, p-value of Chi^2^ (evidence of heterogeneity of intervention effects); I^2^, amount of heterogeneity between trials; Overall P, p-value for significance of effects of interventions; NLC, Nurse-Led Care; PLC, Physician-Led Care; NP, Nurse Practitioner; NP+, Nurse Practitioner with higher degree/courses/specialisation; RN, Registered Nurse. ^*^Andryukhin et al. (2010) [46] reported data at 6 and 18 months and was incorporated accordingly.

Ten trials had sufficient data for meta-analysis, one of which reported different follow-up episodes [46] (Figure 4). The pooled RRs showed a significant reduction in the risk of all-cause mortality with nurse-led care (RRs 0.89, 95% CI 0.84 to 0.96) and no significant heterogeneity between trials (I^2^ = 0%; χ^2^_9df_ = 7.52; p = 0.58). Subgroup analyses by clinical characteristics showed that NPs had an increased effect but less significant than RN/LN in reducing all-cause mortality (NP: RRs 0.76, 95% CI 0.60 to 0.96; RN/LN: RRs 0.92, 95% CI 0.85 to 0.98). Although NPs+ showed an increased estimate, the CIs were wide and less significant (RR 0.19, 95% CI 0.04 to 0.85). The estimate increased in studies of on-going care, non-urgent visits and longer (at least 12 months) follow-up episodes but the CIs and significance remained the same. The effect disappeared in trials of single contact, urgent care visits (n = 1) and shorter (less than 12 months) follow-up episodes. The estimate increased, although with wider CIs and less significance, in trials with inadequate allocation concealment (RRs 0.73, 95% CI 0.58 to 0.91) and in trials with at least 20% attrition (RRs 0.90, 95% CI 0.83 to 0.97). On the other hand, the estimate decreased, with reduced significance, in trials of adequate concealment and trials of less than 20% attrition and disappeared in small trials (N < 200, RR 0.54, 95% CI 0.21 to 1.36). Heterogeneity between trials remained non-significant in all subgroups although low heterogeneity was introduced in trials with less than 20% attrition and smaller trials. The funnel plot was asymmetrical showing five trials falling to the left (nurse-led care with fewer events), two on the right and three on the line of no effect. Data that could not be pooled showed a significantly lower cumulative rate of all-cause mortality and a marginal significance in the cumulative rate of mortality due to coronary/non-fatal myocardial infarction with nurse-led care at 56.4 months [44] (Additional file 1: Table S7). Qualitative data reported to have no documentation of death after 12 months follow-up [36].

#### Sensitivity analyses

In the meta-analyses (Figures 2, 3 and 4), excluding the studies in which nurses had full clinical autonomy or from which this information was not obtainable did not critically alter the estimates (Additional file 1: Table S6). The small non-significant amount of heterogeneity in the meta-analysis of hospital admissions was attributable to a small study which favoured physician-led care but had wide CIs. Excluding quasi-RCTs or cluster RCTs from the meta-analyses of patient satisfaction and mortality slightly reduced the pooled estimate but did not alter the direction of effects and the findings remained significant.

#### Random-effects meta-analyses

Meta-analyses using a RE model showed the same direction of effect. The pooled estimates and heterogeneity remained significant. Patient satisfaction showed an increased estimate although wider CIs (SMD 0.31, 95% CI 0.12 to 0.514, p = 0.002; I^2^ = 91%; χ^2^_6df_ = 65.97; p < 0.0001). Hospital admissions (RRs 0.77, 95% CI 0.63 to 0.94, p = 0.01; I^2^ = 7%; χ^2^_4df_ = 4.30; p = 0.37) and mortality (RRs 0.90, 95% CI 0.84 to 0.96, p = 0.002; I^2^ = 0%; χ^2^ _9df_ = 7.52; p = 0.58) showed very similar estimates of a more modest but yet significant effect.

#### Quality of life

**Figure 5:**
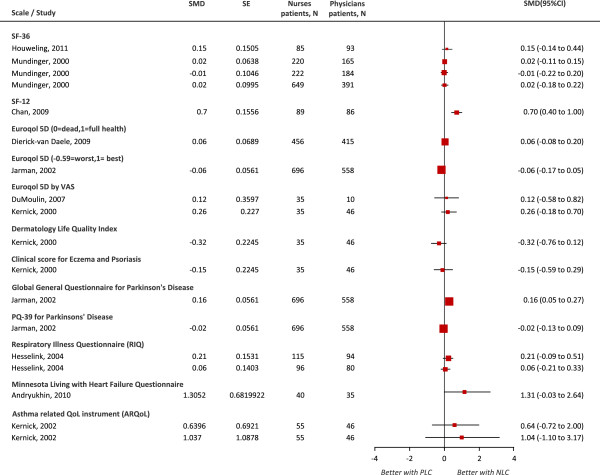
**Comparison of individual trial estimates of the effect of physician-nurse substitution on Quality of Life. **Legend. A pooled estimate was not possible due to the various scales used, grading scores and measurements**.** SMD, standard mean difference; SE, standard error; N, total number of patients per group; CI, confidence interval; NLC, Nurse-Led Care; PLC, Physician-Led Care.

Four [27-29,37] of the thirteen [17,22,23,27-30,33,37,39,42,44,46] trials with measures on QoL used both disease-specific and generic scales of functional health and well-being. Other seven [17,22,23,30,39,42,44] used only generic scales and two used only disease-specific scales [33,46]. Due to the different scales, grading scores and measurements, we decided not to combine trials in a pooled analysis (Figure 5). Comparison of the individual estimates of trials using generic scales showed nurse-led care significantly improved QoL scores with the SF-12 at 6 months (SMD 0.70, 95% CI 0.40 to 1.00) and with the Global General Questionnaire for Parkinson’s Disease at 24 months (SMD 0.16, 95% CI 0.05 to 0.27). Estimates from trials using the SF-36 and Euroqol did not reach significance although some favoured nurse-led care. Trial estimates [27,33,46] using disease-specific scales at 4, 6, 12, and 24 months favoured nurse-led care but were not significant. Four trials reported better scores with nurse-led care in various individual dimensions of the ARQoL, SF-36 and RIQ questionnaires but the overall score was not significant at two weeks [45] or not sustained at least 12 month thresholds [27,33,44] except for patients with incontinence for whom better scores of individual dimensions at 6 months persisted at 12 months or reached a significant overall score (reported p < 0.05) [37]. Qualitative data based on generic scales reported significance (general health questionnaire) [23] or non-significance (SF-36, EQ5D-VAS) in the overall score at 0.5 [17] or at 4 [27] months.

#### Costs

**Figure 6:**
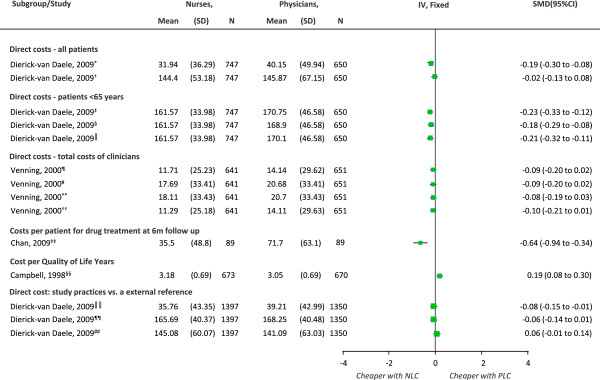
**Comparison of individual trial estimates of the effect of physician-nurse substitution on cost of care. **Legend. A pooled estimate was not possible due to the variety in approaches, currency and indicators used to value resources and to calculate costs. Abbreviations: EUR, Euro; GBP, pound sterling; DCC, direct costs for consultations; DPCC, direct and productivity costs for consultations; general physicians (GP); EMP, employment; EMPO, employment by others; LoC, length of consultations; TCT, total consultation time; TP, time to prescribe; FTF, face-to-face; TT, total time; SD, standard deviation; N, total number of patients per group; SMD, standard mean difference; CI, confidence interval; NLC, Nurse-Led Care; PLC, Physician-Led Care. ^*^EUR; DPCC: GPs salary in EMP and EMPO; p = 0.65**. **^†^EUR; DCC based on resource use, follow-up, LoC /salary; p = 0.0005**. **^‡^EUR; DCC: resource use, follow-up, LoC /salary; p = 0.0001**. **^§^EUR; all patients: DPCC: GP salary in EMP and EMPO; p = 0.0009**. **^║^EUR; <65 years: DPCC: GP salary in EMP and EMPO; p < 0.0001**. **^¶^GBP; return consultations, FTF time: NP = TCT - TP (GP signed); GP = TCT - TP; p = 0.11**. **^#^GBP; initial consultations, TT: NP = TCT + TP (GP signed); GP = TCT + TP; p = 0.11. ^**^GBP; initial consultations, FTF time: NP = TCT – TP (GP signed); GP = TCT + TP; p = 0.07**. **^††^GBP; return consultations, TT: NP = TCT – TP (GP signed); GP = TCT + TP; p = 0.16**. **^‡‡^GBP; costs of drugs; p < 0.0001**. **^§§^GBP; mean QALYs at 48 months: SF-36 overall QoL scores; p = 0.0006**. **^║║^EUR; DPCC: resource use, follow-up, LoC and salary; p = 0.09**. **^¶¶^EUR; DC: resource use, follow-up, LoC and salary; p = 0.04**. **^##^EUR; <65 years of age; DPCC: resource use, follow-up, LoC and salary; p = 0.10.

There were six trials [17,23,29,35,42,44] with data on cost and two [31,38] comprehensive economic evaluations. Due to the large variety of approaches used to value the resources and calculate cost we didn’t pool trials in a meta-analysis. Figure 6 shows the comparison of the individual trial estimates. Costs were generally lower with nurse-led care in direct costs including consultations within study practices, for all patients and in patients not yet 65 years old, in study practices (compared to external reference practices) [38] at 0.5 or 12 [23] months, and in treatment costs with both unadjusted and adjusted data at 6 months [42]. On the other hand, the mean cost per quality adjusted life years (QALYs) at the end of 56.4 months and the cost of interventions (clinics and drugs) were significantly higher with nurse-led care in one trial [44]. Another trial showed lower costs with nurse-led care based on face-to-face total cost of clinicians (total consultation time without the time to get prescriptions signed by physicians or time taken to sign a prescription) [17]. The studies also showed no significant differences between nurses and physicians in direct and productivity costs for consultations in all patients at study practices [38], direct and productivity costs for consultations in all patients or for patients not yet 65 years old at study practices (compared to external reference practices) [38], in the costs of care based on either the total time or face-to-face time given by the nurse or physician [17] or other healthcare system costs (hospitals, outpatient attendances and admissions to private hospitals) [44].

Other trials reported lower healthcare costs with nurse-led care at 6 to 56.4 months [23,35,44] and no significant differences between groups in net healthcare costs [29].

## Discussion

Substitution of physicians by nurses is often discussed and widely practiced in many countries, with the aim of satisfying the demands of an aging population and (local) shortages of physicians. Our review showed that the volume of rigorous evaluations is slowly increasing but remains low. In addition, the quality of available research does allow strong recommendations for practice and policy, despite previous proposals [6,7].

In the appraised literature, the nurses assessed a wide variety of conditions and performed various tasks, with different degrees of clinical autonomy and in different settings. Despite this heterogeneity and the substantial methodological limitations, our review suggested that nurse-led care is associated with higher patient satisfaction, lowered overall mortality and lowered hospital admissions. Effects on other outcomes, such as QoL and costs remained inconclusive.

The effect of nurse-led care on hospital admissions and mortality was particularly present in studies of on-going care and non-urgent visits and when nurse practitioners (both NP and NP with higher degree/courses) provided the care. This suggests that trained nurses can effectively provide healthcare to patients with established diseases. However, the effect disappeared (for hospital admissions) or weakened (for mortality) in studies with better or adequate concealment of allocation and in larger studies. The reasons for this surprising and important finding, especially that nurse-led care could lead to reduced mortality, should be addressed in future studies.

Our overall results also showed a highly significant effect of nurse-led care on patient satisfaction although with severe heterogeneity between trials. This finding is consistent with previous reviews [3,4]. Nevertheless, this result should be interpreted with caution. Although the average effect is positive, subgroups of patients reported less positive views. Our results suggest this variability may be due to nurses’ roles or study size, which may be associated with other factors (such as degree of clinical autonomy). The effect disappeared when we considered only the trials based on on-going care or non-urgent care, and in trials with longer follow-up episodes (at least 6 months), but these subgroups included two trials only. Surprisingly, patient satisfaction was higher with general nurses (as compared to NPs or NPs with higher degree/extra courses), but the two very small studies showing this effect addressed tasks for very special conditions such as incontinence and family planning. This finding fits in with previous research which showed that patients appreciate nurses’ involvement especially in education and counselling [47,48].

The results on QoL were difficult to interpret due to heterogeneous reporting of outcomes and the data that were scattered across different scales with outcome measurements at variable follow-up time intervals. Only a few trials used both generic and disease-specific scales with primarily one trial per scale. There was a potential increase in QoL scores with nurse-led care, when health status was evaluated using generic scales, or for specific conditions (e.g. heart failure, Parkinson’s Disease) but the effect was not significant or not sustained at length (at least 12 months) or it was contradicted by data from the same studies [28]. Similarly, there were some effects of lower costs with nurse-led care, but the reported data used different approaches to value the resources and to calculate costs in only a few trials and economic evaluations.

### Methodological appraisal of included studies

We identified several significant limitations in the current evidence which should be considered in future research. The trials included were highly heterogeneous in terms of tasks, settings, collection and reporting of outcome measurements. There is a considerable amount of data that are reported in descriptive accounts only, limiting both their pooled validity and the interpretation of their results. Additionally, many studies failed to report some important statistical information (e.g. sample sizes, mean scores, SDs) required to calculate trial estimates and to integrate them in a meta-analysis.

No study fulfilled the set of methodological quality criteria assessed, despite widely available guidelines for RCTs. Trials of lower methodological quality (small study, at least 20% attrition and lack/unclear allocation concealment) tended to inflate the results and only less than 50% of the trials maintained the least target sample required to achieve power, which makes results less trustworthy. The most probable small study bias affecting the effect sizes are the results of small negative studies which are generally less likely to be published than small studies with positive results (i.e. publication bias). Blinding (clinicians, patients and outcome assessors) was reported in only a few trials and we don’t rule out the possibility that patient satisfaction, a subjective outcome, may have been especially positively affected by this. The trials consisted of follow-up episodes of variable length (0.5 to 122.4 months) which may have limited the true effect of care especially in multi-morbid or serious illnesses. Our analyses partly explained the reasons for heterogeneity where this was present but several other variables, which we could not account for, may have also caused this. Patients’ perception and evaluation of satisfaction may be inherently subjective due to socio-demographic differences, experiences from previous care, the physical environment, and patient-care provider interactions. Therefore, measurements of outcome using validated tools are preferred. Of the trials appraised, less than 50% used validated questionnaires for patient satisfaction.

We also identified a lack of trials of cluster randomisation. Although these may be more complex in design, if accounted for all key factors including clustering effect, appropriate sampling and analyses, cluster RCTs could add important value to the current evidence.

Surprisingly, there is a dearth of economic data. The little evidence available on the cost of physician-nurse substitution relies on results which are mainly based on direct costs and use variable approaches. The more recent literature reports more economic data, but it seems difficult to integrate these results especially because cost evaluations differ across countries and thus in cost measurements. We found only two publications [31,38] providing economic data related to three of the included trials. Despite continued claims of substituting physicians by nurses based on healthcare costs, the evidence can only suggest that substitution is cost neutral. Therefore, as suggested in a recent systematic review of economic evaluations [49], to meaningfully place the costs and consequences of substitution in the context of healthcare, studies should address all types of costs. Relevant and appropriate data should be generated by means of a systematic collection of economic measures, and specific rules for cost data estimations should also be defined and followed.

More intensive implementation could enhance the outcomes of nursing care, but most studies do not provide the necessary information. In the evaluated studies, the assumption is that nurses possess the competence required for substituting physicians, but the level of substitution does not seem equal among studies. While the level of training may be a critical factor for an effective outcome, the studies report incomplete descriptions of nurses’ roles and competencies. The level of clinical autonomy in nurses does not seem consistent with the level of training and the tasks performed. Also, nurses still require support or communication with the physician for various tasks. It seems then that the level of qualification and training required to carry out substitution requires yet a better definition of practice boundaries including a classification of tasks. Better criteria conceptualised to define nurses’ roles and responsibilities are needed. In addition, the various differences between countries’ definitions and their organisation of nurse care should be taken into account. Lastly, more than half of the evidence reviewed (62.5%) has been conducted in Europe, mainly the UK and the Netherlands.

It is apparent that there is much room for primary studies that include larger numbers of patients, methodologically more rigorous in terms of quality, comprehensive in terms of data and statistical methods and with longer follow-up episodes. Furthermore, in order to gain a better understanding of substitution, future research should map a wider range of nurses, the various levels of training and clinicians’ characteristics, which are provided in many countries. As suggested previously [50], each method of skill-mix may have its own strengths and weaknesses. The implementation of methodologies aiming at the standardisation of skill-mix studies could support a sound assessment such that health sector reform may also benefit from the publication of evidence.

### Strengths and limitations of the review

Our review updates and extends earlier systematic reviews [3,4] and benefits from a thorough assessment of RCTs, in which the nurse acted as the main figure of care. It also presents (where available) the results by nurses’ roles. Having used the fixed effect model, we can only make inferences about the studies included in the meta-analyses performed here. We only included RCTs because these are at a lower risk of bias and allow for the identification of causal relationships. Although non-randomised trials may overestimate the benefits of nurse-led care it would be recommended to scrutinise the current evidence with such designs. These may not only provide an opportunity for an update but also allow for the collection of data from long term (more than 12 months) follow-up designs which may consist of larger sample sizes. We only included publications in English. We did however screen the reference lists of relevant reviews (some in foreign languages) and searched the reference lists of all included studies. We did not contact authors for further information nor did we search for grey literature. A further limitation is that it was often difficult to understand in detail what role and responsibilities nurses had, when substituting physicians. In many cases, they remain embedded in patient care teams that also involved physicians.

## Conclusion

The slowly growing number of studies, assessing substitution of physicians by nurses is still substantially limited by methodological deficiencies. Also, the current evidence belongs to a small selection of healthcare systems lacking good quality data*.* Nevertheless, nurse-led care seems to have a positive effect on hospital admissions and mortality. This important finding should be confirmed and the determinants of this effect should be assessed in future studies. Before implementing new changes in the delivery of healthcare, further, larger and more methodically rigorous primary research should address the quality of the data on both health outcomes and costs. Primary research should also differentiate between types of nurses, qualifications and tasks. In particular, we recommend considering the role of multidisciplinary teams in which nurses are embedded, also when substituting physicians in specific clinical tasks.

## Competing interests

The authors declare no competing interests.

## Authors’ contributions

NAMG: design and analyses of the study; conceptualisation of the study; design and formulation of search strategies; screening of titles, abstracts and full texts; acquisition of the data; planning of the analysis and interpretation of data; quality assessment; wrote and revised the manuscript. SD: contribution to the design and conceptualisation of the study; screening of titles, abstracts and full texts; acquisition of data and quality assessment. RT: contribution to the design and conceptualisation of the study; support clinical input on eligibility of studies; and on extraction of reported clinical data. FH-G: contribution to the design and conceptualisation of the study; screening of titles, abstracts and full texts; quality assessment and data extraction. SM: contribution to the consensus of data. MW: contribution with revision of the manuscript. TR: senior supervision of the study, oversaw the development and methodology of the review; input on eligibility of studies and on the interpretation of the data; revision of the manuscript. All authors read and approved the final version of the manuscript to be published.

## Pre-publication history

The pre-publication history for this paper can be accessed here:

http://www.biomedcentral.com/1472-6963/14/214/prepub

## Supplementary Material

Additional file 1 **List of Tables supporting the results of studies included in review. Table S1:** PRISMA Checklist. **Table S2:** Search strategy in Ovid Medline. **Table S3:** Studies excluded with reasons for exclusion based on appraisal of full text articles. **Table S4:** Characteristics of participants and interventions in the included studies. **Table S5:** Summary of nurses’ roles, clinical autonomy and type of care. **Table S6:** Sensitivity analyses. **Table S7:** Individual trial estimates from data not combined in meta-analyses. Click here for file
