# Supplementary material for: Substitution of physicians by nurses in primary care: a systematic review and meta-analysis
Source: BMC Health Serv Res. 2014 May 12;14:214. doi: 10.1186/1472-6963-14-214 (PMC4065389; doi:10.1186/1472-6963-14-214)
Supplement: Additional file 1 — List of Tables supporting the results of studies included in review. Table S1: PRISMA Checklist. Table S2: Search strategy in Ovid Medline. Table S3: Studies excluded with reasons for exclusion based on appraisal of full text articles. Table S4: Characteristics of participants and interventions in the included studies. Table S5: Summary of nurses’ roles, clinical autonomy and type of care. Table S6: Sensitivity analyses. Table S7: Individual trial estimates from data not combined in meta-analyses. [file 1472-6963-14-214-S1.doc]

Additional file

List of Tables supporting the results of studies included in review.

Table S1: PRISMA Checklist.

Table S2: Search strategy in Ovid Medline.

Table S3: Studies excluded with reasons for exclusion based on appraisal of full text articles.

Table S4: Characteristics of participants and interventions in the included studies.

Table S5: Summary of nurses’ roles, clinical autonomy and type of care.

Table S6: Sensitivity analyses.

Table S7: Individual trial estimates from data not combined in meta-analyses.

**Additional file, Table S1: PRISMA Checklist.**

| **Section/topic** | **#** | **Checklist item** | **Reported on page #** |
| --- | --- | --- | --- |
| **TITLE** | | |  |
| Title | 1 | Identify the report as a systematic review, meta-analysis, or both. | 1 |
| **ABSTRACT** | | |  |
| Structured summary | 2 | Provide a structured summary including, as applicable: background; objectives; data sources; study eligibility criteria, participants, and interventions; study appraisal and synthesis methods; results; limitations; conclusions and implications of key findings; systematic review registration number. | 2 |
| **INTRODUCTION** | | |  |
| Rationale | 3 | Describe the rationale for the review in the context of what is already known. | 4 |
| Objectives | 4 | Provide an explicit statement of questions being addressed with reference to participants, interventions, comparisons, outcomes, and study design (PICOS). | 4 |
| **METHODS** | | |  |
| Protocol and registration | 5 | Indicate if a review protocol exists, if and where it can be accessed (e.g., Web address), and, if available, provide registration information including registration number. | 4 |
| Eligibility criteria | 6 | Specify study characteristics (e.g., PICOS, length of follow-up) and report characteristics (e.g., years considered, language, publication status) used as criteria for eligibility, giving rationale. | 4 |
| Information sources | 7 | Describe all information sources (e.g., databases with dates of coverage, contact with study authors to identify additional studies) in the search and date last searched. | 5 |
| Search | 8 | Present full electronic search strategy for at least one database, including any limits used, such that it may be repeated. | 5, Additional Table S2 |
| Study selection | 9 | State the process for selecting studies (i.e., screening, eligibility, included in systematic review, and, if applicable, included in the meta-analysis). | 6 |
| Data collection process | 10 | Describe method of data extraction from reports (e.g., piloted forms, independently, in duplicate) and any processes for obtaining and confirming data from investigators. | 5-6 |
| Data items | 11 | List and define all variables for which data were sought (e.g., PICOS, funding sources) and any assumptions and simplifications made. | 5 |
| Risk of bias in individual studies | 12 | Describe methods used for assessing risk of bias of individual studies (including specification of whether this was done at the study or outcome level), and how this information is to be used in any data synthesis. | 5 |
| Summary measures | 13 | State the principal summary measures (e.g., risk ratio, difference in means). | 6 |
| Synthesis of results | 14 | Describe the methods of handling data and combining results of studies, if done, including measures of consistency (e.g., I^2^) for each meta-analysis. | 6 |
| Risk of bias across studies | 15 | Specify any assessment of risk of bias that may affect the cumulative evidence (e.g., publication bias, selective reporting within studies). | 6-7 |
| Additional analyses | 16 | Describe methods of additional analyses (e.g., sensitivity or subgroup analyses, meta-regression), if done, indicating which were pre-specified. | 6-7 |
| **RESULTS** | | |  |
| Study selection | 17 | Give numbers of studies screened, assessed for eligibility, and included in the review, with reasons for exclusions at each stage, ideally with a flow diagram. | 7, Figure 1 |
| Study characteristics | 18 | For each study, present characteristics for which data were extracted (e.g., study size, PICOS, follow-up period) and provide the citations. | 7-8, Table 1 |
| Risk of bias within studies | 19 | Present data on risk of bias of each study and, if available, any outcome level assessment (see item 12). | 9, Table 2 |
| Results of individual studies | 20 | For all outcomes considered (benefits or harms), present, for each study: (a) simple summary data for each intervention group (b) effect estimates and confidence intervals, ideally with a forest plot. | 9, Figures 2-6, Additional file, Table S6 |
| Synthesis of results | 21 | Present results of each meta-analysis done, including confidence intervals and measures of consistency. | 9-14 |
| Risk of bias across studies | 22 | Present results of any assessment of risk of bias across studies (see Item 15). | 9-12, Table 2, Figures 2-4 |
| Additional analysis | 23 | Give results of additional analyses, if done (e.g., sensitivity or subgroup analyses, meta-regression [see Item 16]). | 12, Additional file, Table S5 |
| **DISCUSSION** | | |  |
| Summary of evidence | 24 | Summarize the main findings including the strength of evidence for each main outcome; consider their relevance to key groups (e.g., healthcare providers, users, and policy makers). | 14 |
| Limitations | 25 | Discuss limitations at study and outcome level (e.g., risk of bias), and at review-level (e.g., incomplete retrieval of identified research, reporting bias). | 18 |
| Conclusions | 26 | Provide a general interpretation of the results in the context of other evidence, and implications for future research. | 19 |
| **FUNDING** | | |  |
| Funding | 27 | Describe sources of funding for the systematic review and other support (e.g., supply of data); role of funders for the systematic review. | 20 |

*From:*  Moher D, Liberati A, Tetzlaff J, Altman DG, The PRISMA Group (2009). Preferred Reporting Items for Systematic Reviews and Meta-Analyses: The PRISMA Statement. PLoS Med 6(6): e1000097. doi:10.1371/journal.pmed1000097

**Additional file, Table S2:** Search strategy in Ovid Medline*.

| **#** | **Searches** | **Results** |
| --- | --- | --- |
| 1 | exp General Practice/ or exp Primary Health Care/ or exp Private Practice/ or Family Physicians/ or general practitioners/ or physicians, primary care/ or exp geriatrics/ or Geriatric Assessment/ or exp pediatrics/ | 223536 |
| 2 | exp Nursing Care/ or Primary Nursing/ or Community Health Nursing/ or Family Nursing/ or Nursing, Practical/ or Advanced Practice Nursing/ or exp Geriatric Nursing/ or exp Pediatric Nursing/ | 129054 |
| 3 | exp Ambulatory Care/ or ambulatory care facilities/ or community health centers/ or maternal-child health centers/ or outpatient clinics, hospital/ or pain clinics/ or surgicenters/ | 75627 |
| 4 | (primary adj2 (health?care$ or care$ or medic$)).ti,ab,hw. | 96885 |
| 5 | ((family adj2 (physician$ or doctor or practitioner or practice or internist or medic$)) or (general adj2 (internist or physician$ or doctor or practitioner or practice or medic$ or care$ or health$care$)) or (personal adj2 (doctor or physician$)) or (physician$ adj2 (practitioner or practice)) or (internal adj medicine) or geriatri$ or paediatri$ or pediatri$).ti,ab,hw,mp. | 459697 |
| 6 | ((community or ambulatory or shared) adj4 (care$ or health$care) adj4 (facility or facilities or service$ or cent$ or clinic$)).ti,ab,hw. | 15372 |
| 7 | or/1-6 | 713736 |
| 8 | exp Physician Assistants/ or nurse clinicians/ or nurse practitioners/ | 23718 |
| 9 | (nurs$ adj2 (family or primary or care$ or practitioner or practice or clinic$ or regist$ or specialist$ or leader or consultant$ or physician$ or expert or district or advanced or assessment or visit$ or geriatri$ or paediatri$ or pediatri$)).tw,mp. | 150356 |
| 10 | ((nurs$ adj2 appropriately adj trained) or (nurs$ adj2 community adj2 health adj2 care) or (nurs$ adj2 first contact) or (assistan$ adj2 (physician$ or medic$ or health$care$)) or (clinic$ adj2 support) or (clinic$ adj2 (nurse$led or nurse led))).ti,ab,hw. | 20215 |
| 11 | or/8-10 | 175262 |
| 12 | Nurse's Practice Patterns/ | 553 |
| 13 | delegation, professional/ | 360 |
| 14 | professional autonomy/ | 8143 |
| 15 | Clinical Competence/ | 58713 |
| 16 | exp Professional Role/ | 61186 |
| 17 | 12 or 13 or 14 or 15 or 16 | 120249 |
| 18 | (((substitut$ or transfer$ or swap or replac$) adj3 (((doctor$ or GP or GPs or physician$ or practi$ or general) adj2 practitioner$) or job or role or task$ or skill$ or perform$ or responsibility or autonom$)) or ((delegat$ or supervis$) adj5 (responsibility or performance$ or role$ or job or tasks)) or (autonom$ adj (professional or responsibility or self$regulation)) or (clinical adj skill$ adj competence) or (((skill$mix or skill$) adj mix$) or skill$) or (role$ adj4 (advance or chang$ or enhanc$ or expan$ or transfer$)) or (team$ adj4 (patient care or multidisciplinary or cooperation) adj4 autonom$)).ti,ab,hw. | 150274 |
| 19 | 17 or 18 | 254445 |
| 20 | 7 and 11 and 19 | 14767 |
| 21 | (letter or letter$).pt,sh. or (editorial or historical article or anecdote or commentary or note or case report$ or case study).pt. or (editorial or historical article or anecdote or commentary or note or case report$ or case study).pt. or (animal studies or animals, laboratory or experimental animal or animal experiment or animal model or rodentia or rodents or rodent).sh. | 2816007 |
| 22 | (randomi?ed controlled trial or controlled clinical trial).pt. or (randomi?ed or placebo or randomly or trial or groups).ab. | 1748897 |
| 23 | exp cluster analysis/ or cross-over studies/ or ((cluster$ adj2 random$) or (communit$ adj2 intervention$) or (communit$ adj2 random$)).mp. | 74125 |
| 24 | ((non$equivalent adj3 control$) or posttest$ or post test$ or post-test$ or pre test$ or pretest$ or pre-test$ or quasi-experiment$ or quasi experiment$ or quasiexperiment$ or timeseries or time series or time-series or (time adj2series adj2 analysis) or (interrupted adj2 time adj2series)).mp. | 34416 |
| 25 | 22 or 23 or 24 | 1807744 |
| 26 | 25 not 21 | 1780623 |
| 27 | 20 and 26 | 1376 |
| 28 | limit 27 to humans | 1348 |

**Legend.**

*Similar search strategies were performed and run in EMBASE, The Cochrane Library of Systematic Reviews and CINAHL and include specific search filters for RCTs.

**Additional file, Table S3:** Studies excluded with reasons for exclusion based on appraisal of full text articles.

| **Item** | **References of studies** | **Reason for exclusion** |
| --- | --- | --- |
|  | Blanchard MR, Waterreus A, Mann AH (1995) The effect of primary care nurse intervention upon older people screened as depressed. Int J Geriatr Psychiatry 10: 289-298. | Cohort study and multidisciplinary team approach. |
|  | Blanchard MR, Waterreus A, Mann AH (1999) Can a brief intervention have a longer-term benefit? The case of the research nurse and depressed older people in the community. Int J Geriatr Psychiatry 14: 733-738. | Nurse working in close collaboration with other clinicians. |
|  | Cave AJ, Wright A, Dorrett J, McErlain M (2001) Evaluation of a nurse-run asthma clinic in general practice. Primary Care Respiratory Journal 10: 65-68. | Not an intervention comparison between nurses and physicians. |
|  | Krein SL, Klamerus ML, Vijan S, Lee JL, Fitzgerald JT, et al. (2004) Case management for patients with poorly controlled diabetes: a randomized trial. Am J Med 116: 732-739. | Nurse working in close collaboration with other clinicians based on a chronic care model. |
|  | Lapointe F, Lepage S, Larrivee L, Maheux P (2006) Surveillance and treatment of dyslipidemia in the post-infarct patient: can a nurse-led management approach make a difference? Can J Cardiol 22: 761-767. | Intervention (telephone) not of interest for this review and not part of usual care interventions of physicians. |
|  | Leenders F, Beusmans G, Swerts H, editors (2006) A practice nurse for patients with cardiovascular disease, an explorative study. | Report in Dutch. Version of article in English was not found. |
|  | Lewis CE, Resnik BA, Schmidt G, Waxman D (1969) Activities, events and outcomes in ambulatory patient care. N Engl J Med 280: 645-649. | Observational study and outcomes not of interest for this review. |
|  | Sackett DL, Spitzer WO, Gent M, Roberts RS (1974) The Burlington randomized trial of the nurse practitioner: health outcomes of patients. Ann Intern Med 80: 137-142. | No real substitution. At least 30% of patients in both groups were seen by the physicians at the end of study and data was not split into mutually exclusive groups. |
|  | Spitzer WO, Sackett DL, Sibley JC, Roberts RS, Gent M, et al. (1974) The Burlington randomized trial of the nurse practitioner. N Engl J Med 290: 251-256. | No real substitution. At least 30% of patients in both groups were seen by the physicians at the end of the study and data was not split into mutually exclusive groups. |
|  | Tonstad S, Alm CS, Sandvik E (2007) Effect of nurse counselling on metabolic risk factors in patients with mild hypertension: a randomised controlled trial. European Journal of Cardiovascular Nursing 6: 160-164. | No real substitution. In both experimental and control groups the nurse provides interventions and different stages of care. |
|  | Van Son L, Vrijhoef, H. (2004) Supporting the general practitioner. A randomized controlled trial investigation the effects of a practice nurse on asthma, COPD, and diabetes. Huisarts en wetenschap: 15-21. | Report in Dutch. Version of article in English was not found. |
|  | Williams KS, Assassa RP, Cooper NJ, Turner DA, Shaw C, et al. (2005) Clinical and cost-effectiveness of a new nurse-led continence service: a randomised controlled trial. Br J Gen Pract 55: 696-703. | Nurse working in close collaboration with other clinicians. Control group received care from nurses, physicians and specialists and data was not split into mutually exclusive groups. |

**Additional file, Table S4: Characteristics of participants and interventions in the included studies.**

| **Study** | | **Participants** | | **Intervention** | | |
| --- | --- | --- | --- | --- | --- | --- |
| **Location, first author and year of publication, duration of study** | **Design, period**^*^ | **Included** | **Excluded** | **Nurses’ role/competency** | **Clinical autonomy** | **Follow-up, months** |
| ZA 2 | cRCT (cohort 2), 2008-2010. | Adults with HIV who received ART for at least six months with ongoing treatment at the time of enrolment; clinics with more than 100 eligible patients used a random sample taken electronically (proportional to the total number of eligible patients); in other clinics all eligible patients were included. | Patients who did not return to their clinic after enrolment or who were potentially exposed to the intervention; deaths before trial started or were relocated to another clinic. | Nurse-led care based on African guidelines. Middle nurse managers trained to assume responsibility for ART: i.e. assess and prepare patients, initiate, monitor and prescribe ART or referral to physicians for ART initiation and re-prescriptions. Physicians referred patients diagnosed with HIV to nurse-led clinic to establish eligibility for ART. | For phase 2 and phase 3: same as in cohort 1. | 18 |
| Fairall, 2012 [36] |
| Study duration, months: 29 |
| ZA 1 | cRCT (cohort 1), 2008-2010. | Adults with HIV participating in an ART program who had CD4 counts of ≤350/µl and had not yet started ART: either eligible for ART (if CD4 counts were ≤200 cells per μL) or likely to become eligible during the trial (if CD4 counts were 201-350 cells per μL). | Patients who did not return to their clinic after enrolment because they needed to visit a clinic more than once to initiate ART after obtaining CD4 results; who started ART before trial started, who deaths before trial started or were relocated to another clinic. | Nurse-led care based on African guidelines. Middle nurse managers trained to assume responsibility for ART: i.e. assess and prepare patients; initiate, monitor and prescribe ART or referral to physicians for ART initiation and re-prescriptions. Physicians referred patients diagnosed with HIV to nurse-led clinic to establish eligibility for ART. | For phase 2: nurses assumed responsibility for repeating ART prescriptions in stable (median, m: 3.5, range: 1-35) patients. For phase 3: nurses assumed responsibility for initiating ART in selected patients (median, m: 30.5, range: 0-32) and nurses referred patients who did not meet above criteria. | 16-18 |
| Fairall, 2012 [36] |
| Study duration, months: 29 |
| NL 6 | RCT, period nr. | Patients under treatment and under medication for diabetes mellitus type two, with HbA1c measurements within the last three years. | Patients with diabetes mellitus type two not being treated in primary care setting, inability to participate because of age, comorbidities or -in the opinion of the general practitioner- whoever was not willing to return for follow-up. | Nurse-led care based on guidelines. Practice nurse with one week training in diabetes mellitus to manage transferred patients based on guidelines. | Full responsibility. | 14 |
| Houweling, 2011 [30] |
| Study duration, months:14 |
| NL 5 | RCT, 2006-2008. | Patients 6-16 years old with moderate and stable asthma, in treatment of inhalative corticosteroids at least nine months before the start of the study, informed consent. | Patients not able to perform lung function tests, or who had other chronic diseases. | Asthma nurse to manage patients based on guidelines. | Nurses needed support from/or short communication with paediatrician. | 24 |
| Kuethe, 2011 [25] |
| Study duration, months: 24 |
| RU 1 | RCT, 2006-2009. | Patients of at least 50 years of age with heart failure with preserved ejection fraction, informed consent. | Patients with blood pressure of <90/60 mmHg or >160/100 mmHg, under optimal antihypertensive therapy, acute coronary syndrome within previous six months, significant valvular stenosis, insulin diabetes mellitus dependent, confirmed chronic obstructive pulmonary disease, conditions limiting participation in the rehabilitation (see reference for more details). | Nurse-led care based on Russian National guidelines. Nurses with special degree in patient education obtained in joint course: patient education, treatment and exercise training information and counselling. | Prescription of medication and non-pharmacological measures (diet, alcohol intake, weight reduction, smoking cessation, activity and exercise training) provided by physician. | 6, 18 |
| Andryukhin, 2010 [46] |
| Study duration, months: 36 |
| NL 4 | RCT, 2006-2007. | Patients 30-74 years of age, with cardiovascular disease or hypertension and/or hypercholesterolemia, with at least 10% in 10-year risk of cardiovascular disease; risk due to systolic blood pressure of ≥140 or total cholesterol of ≥6.5 mmol/l within the previous six months. | Patients visiting specialist in cardiovascular disease more than once per year, diabetes mellitus, severe comorbidities. | Nurse-led care based on Dutch guidelines for cardiovascular risk management. Advance practice nurse managed cardiovascular risk including primary and secondary prevention. | nr. | 12 |
| Voogdt-Pruis, 2010 [16] |
| Study duration, months: 12 |
| NL 3 | RCT, 2006. | Patients with common complaints aged ≥16 who sought general practitioner for initial consultation. | Patients unregistered in practice, language or reading problems, or with reason for appointment not provided | Practice nurse with Master degree in Advance Nursing, trained in common complaints to manage patients based on guidelines (assess symptoms, perform PE and diagnosis; decisions on further treatment, prescribing, referrals to 1ry and 2ry services, ordering clinical tests and investigations). | Prescriptions and referrals had to be validated by physician. | 0.5 |
| Dierick-Van Dale, 2009 [39] |
| Study duration, months: 6 |
| UK 9 | RCT, 2002-2004. | Patients with mild gastro-oesophageal reflux disease or moderate gastritis referred to gastroscopy for evaluation. | Patients with sinister symptoms (dysphagia, vomiting, anaemia, rapid weight loss, history of gastric surgery, severe gastroscopic findings e.g. peptic ulcer, tumour, esophagitis grade C/D, Barrett’s oesophagus, anatomical abnormality). | Gastrointestinal nurse practitioner to manage Dyspepsia based on guidelines and to run follow-up clinic for consultations following gastroscopy. | Authorised to adopt treatment according to guidelines and perform specific tests (e.g. breath urea, barium meal). | 6 |
| Chan, 2009 [42] |
| Study duration, months: 24 |
| NL 2 | cRCT, period nr. | Female community-dwelling patients, older than 18 years with any form of incontinence, presenting with urinary incontinence for the first time, informed consent. | Patients with bacteriuria, with post-void residual urine volume of more than100 ml, who gave birth within three months before study, with bladder cancer, renal disease, uterus prolapse or past introitus. | Registered nurse specialist in incontinence to provide care based on protocols: management and patient education gave advice on lifestyle, treatment and use of pads; and referred patients to urologist. | Reporting of findings to physician after each visit; urinalysis completed by physician. | 12 |
| Du Moulin, 2007 [37] |
| Study duration, months: nr |
| US 6 | RCT, period nr. | Patients of at least 18 years of age with diabetes mellitus type two. | nr. | Diabetes nurse to manage patients based on guidelines (evaluation/examination; identification/discussion of short/long term goals; action plan, proactive/continuous follow-up; communicating results to patient and physician; blood samples for urine analysis). | Nurses received support from physicians as the physicians of record to discuss patients’ records; nurses also developed an action plan together with the physicians. | 6 |
| Hiss, 2007 [32] |
| Study duration, months: 6 |
| NL 1 | RCT, 2000-2001. | Patients with asthma, chronic obstructive pulmonary disease or mixed disease aged 16 to 75 years, with symptoms (cough, phlegm or dyspnoea) within year before study, with current use of chronic obstructive pulmonary disease or asthma medication. | Patients with presence of other pulmonic disease, terminal disease. | Physician’s assistants to manage patients based on semi-structured protocols: Asthma and chronic obstructive pulmonary disease patient education. | No (details nr). | 24 |
| Hesselink, 2004 [33] |
| Study duration, months: 36 |
| UK 8 | RCT, 2000-2001. | Patients with diabetes mellitus type two, previous diagnosis of hypertension, or who were in receipt of blood pressure lowering treatment. | Patients with life-threatening comorbidities requiring intensive management. | Hypertension nurse care based on clinical guidelines. | No (details nr). | 6 |
| Denver, 2003 [40] |
| Study duration, months: 6 |
| UK 7 | RCT, 1996-1999. | Patients with Parkinson’s Disease taking one or more anti-Parkinson drugs, informed consent. | Patients younger than 17 years of age, severe mental illness, sufficient cognitive impairment. | Community nurse with a course in Parkinson’s Disease: advised physicians, provided patient counselling and education, treatment information and monitoring; reporting to physicians, instigating respite, day hospital care and discharge; assessment of patient social security, liaison with multidisciplinary primary care teams for ongoing assessment and therapy. | Nurses were under guidance of a nurse manager but had advisory position to physicians with whom patients’ records were discussed. | 24 |
| Jarman, 2002 [29] |
| Study duration, months: 24 |
| UK 6 | RCT, period nr. | Patients with asthma aged 18–25 years. | Patients with previous attendance to nurse asthma clinic. | Nurse with structured training in asthma care to manage patients based on guidelines: assessment, patient education and treatment recommendations. | Prescriptions required a doctor's signature if recommendations were in line with guidelines. | 4 |
| Kernick, 2002 [27] |
| Study duration, months: 4 |
| US 5 | RCT, 1995-1997. | Patients with previous diagnosis of asthma, diabetes mellitus, hypertension, or requesting urgent visits. | Patients having a current primary care provider, planned to leave the area within the following six months, or failed to keep their initial appointment. | Community nurse practitioners provided ambulatory primary care, 24-hr call, made independent decisions for referral to specialists and hospitalisations. | Independent decisions for referrals to specialists and hospitalisations but unclear about the rest e.g. prescriptions. | 6-12 (phase I), 24 (phase II) |
| Mundinger, 2000 [22,24] |
| Study duration, months: 27 |
| UK 5 | RCT, period nr. | Patients with diagnosis of psoriasis or eczema aged 18 to 65 years and a minimum of 3 repeated prescriptions for a topic medication in the past year. | nr. | Practice nurse with training in management of psoriasis and eczema and to deliver care based on dermatology manual: management and prescription of medications. | Prescriptions required a doctor's signature if recommendations were in line with guidelines. | 4 |
| Kernick, 2000 [28] |
| Study duration, months: 4 |
| UK 4 | RCT, period nr. | Patients with diverse complaints requesting same day appointments, informed consent. | Patients seemingly too ill to wait or unable to understand the research, women seeking emergency contraception. | Nurse practitioner with a nurse diploma on care for same day consultations for primary care. | Physicians were always available to prescribe when necessary. | 0.5-1 |
| Kinnersley, 2000 [26] |
| Study duration, months: nr |
| UK 3 | RCT, period nr. | Patients with diverse complaints requesting same day appointments and able to meet time slot saved for study consultation. | Patients with temporary residency permit to area, not yet registered in practice, language or reading problems, too ill, children aged <16 years without company. | Nurse with course at BSc or MSc level: care for same day consultations: book appointments, take history; perform diagnosis, examinations and tests; prescribe and refer patients. | Prescriptions required a doctor's signature. | 0.5 |
| Venning, 2000 [17] |
| Study duration, months: nr |
| UK 2 | RCT, 1998-1999. | Patients with minor illnesses of ≥1 years of age, who requested and were given appointment on the same day, informed consent. | Patients with pregnancy problems, severe chest or abdominal pain, severe breathing problems, vomiting blood, fits or blackouts, psychiatric problems, literacy or language difficulties. | Practice nurse who had no specific experience in seeing patients with minor illnesses but took a course on managing minor illnesses and were piloted before the study: management, history taking, physical examinations, advice and treatment, prescribing and referral. | Prescriptions required a doctor's signature. | 0.5 |
| Shum, 2000 [18] |
| Study duration, months: 4 |
| US 4 | qRCT, 1999-2001. | Patients with undifferentiated conditions and primary care referrals assigned to "any available provider". | Patients with assignment to particular health care professional due triage based on severity of symptoms, patients who had primary care visit within 1 year prior to intervention. | Nurse practitioner who was on staff for at least six months in primary care. | Full responsibility to manage patients. | 12 |
| Hemani, 1999 [34] |
| Study duration, months: 12 |
| UK 1 | RCT, 1995-1996. | Patients with coronary heart disease. | Patients with terminal illness, dementia, housebound patient, explicit request to see general practitioner. | Health visitors, district and practice nurses with training in clinic protocols/guidelines and techniques to facilitate behavioural change: secondary prevention of coronary heart disease. | No full responsibility to manage patients. | 12, 24, 56.4, 122.4 |
| Campbell, 1998 [19-21,41, 43-45] |
| Study duration, months: 6-12 |
| US 3 | RCT, 1980. | Patients requiring family planning, consultation for venereal diseases, acute non-traumatic minor illnesses; age ≥16 years or <16 years in company of a parent seeking care for first time, with illness appropriate to manage for nurse practitioner or medical doctor. | Patients requesting a particular provider. | Registered professional nurse followed standard protocols; had preparation and skills in physical diagnosis, psychosocial assessment, and management of health-illness in primary care: family planning, venereal diseases and acute non-traumatic minor illnesses. | nr. | 0-0.5 |
| Winter, 1981 [15] |
| Study duration, months: 6 |
| US 2 | RCT, 1971. | Patients with undifferentiated conditions with informed consent were referred to nurse clinician. | nr. | Nurse clinicians with training in service delivery including health status, quantity and efficiency of care. | Nurses were authorised to order medication and tests (assumed full clinical responsibility). | ≥6 |
| Flynn, 1974 [35] |
| Study duration, months: 6-12 |
| US 1 | RCT, period nr. | Patients with hypertension, cardiovascular disease, obesity, somatisation problems, rheumatoid or degenerative arthritis. | nr. | Nurses who provided primary source care for at least 1 year before the study to provide care based on guidelines: routine management, schedule appointments and care following standing orders written for patients in each diagnostic class. | The diagnostic class defined the limits for nurses’ autonomy i.e. initiation or alteration of care; patient charts seen by nurse were reviewed daily by 1–2 physicians involved in the project. | 12 |
| Lewis, 1967 [23] |
| Study duration, months: nr |

**Legend.**

Studies are listed by year (y) of publication, in decreasing order.

US, United States; NL, The Netherlands; UK, United Kingdom; ZA, South Africa; RU, Russia; RCT, Randomised Controlled Trial; cRCT, Cluster Randomised Controlled Trial; nr, Not Reported; ART, Antiretroviral Therapy; HbA1c, Haemoglobin; CD4, t-cell surface glycoprotein CD4.

^*^Start and end year when studies were conducted.

**Additional file, Table S5:** Summary of nurses’ roles, clinical autonomy and type of care.

| **Study details** | **Disease** | **Intervention delivered by** | **1^st^C** | **UV** | **OC** | **C, n** | **FCA** | **GDL/PTCL** | **FUP, m** |
| --- | --- | --- | --- | --- | --- | --- | --- | --- | --- |
| ZA 2 Fairall, 2012 [36] | HIV/AIDS. | LN | no | no | yes | >1 | No | GDL | 18 |
| ZA 1 Fairall, 2012 [36] | HIV/AIDS. | LN | no | no | yes | >1 | No | GDL | 16-18 |
| NL 6  Houweling, 2011 [30] | Diabetes Mellitus Type II. | NP | no | no | yes | >1 | Yes | GDL | 14 |
| NL 5  Kuethe, 2011 [25] | Asthma. | NP+ | no | no | yes | >1 | No | GDL | 24 |
| RU 1 Andryukhin, 2010 [46] | Heart Failure with Preserved Ejection Fracture. | NP/LN | no | no | yes | >1 | No | GDL | 6, 18 |
| NL 4  Voogdt-Pruis, 2010 [16] | CVD, Hypertension, Hypercholesterolemia. | NP+ | no | no | yes | >1 | NR | GDL | 12 |
| NL 3 Dierick-Van Dale, 2009 [39] | Common complaints. | NP+ | yes | no | no | 1 | No | GDL | 0.5 |
| UK 9 Chan, 2009 [42] | GORD, moderate Gastritis. | NP+ | no | no | yes | >1 | No | GDL | 6 |
| NL 2 Du Moulin, 2007 [37] | All forms of incontinence. | RN | yes | no | yes | >1 | No | PTCL | 12 |
| US 6 Hiss, 2007 [32] | Diabetes Mellitus. | NP+ | no | no | yes | >1 | No | GDL | 6 |
| NL 1 Hesselink, 2004 [33] | Asthma and COPD. | LN | no | no | yes | >1 | No | PTCL | 24 |
| UK 8 Denver, 2003 [40] | Diabetes Mellitus Type II pre-diagnosed with Hypertension or in receipt of BPLT. | NP+ | no | no | yes | >1 | No | GDL | 6 |
| UK 7 Jarman, 2002 [29] | Parkinson's Disease. | LN | no | no | yes | >1 | No | NR | 24 |
| UK 6 Kernick, 2002 [27] | Asthma | RN/LN | yes | no | yes | >1 | No | GDL | 4 |
| US 5 Mundinger, 2000 [22,24] | Asthma, Diabetes Mellitus, Hypertension, or urgent visits. | NP | yes | yes | yes | >1 | No | NR | 6-12, 24 |
| UK 5 Kernick, 2000 [28] | Psoriasis and Eczema. | NP+ | no | no | yes | >1 | No | GDL | 4 |
| UK 4 Kinnersley, 2000 [26] | Diverse complaints. | NP | yes | yes | no | 1 | No | NR | 0.5-1 |
| UK 3 Venning, 2000 [17] | Diverse complaints: e.g. minor injuries, respiratory complaints. | NP/NP+ | yes | yes | no | 1 | No | NR | 0.5 |
| UK 2 Shum, 2000 [18] | Acute minor illnesses. | NP | yes | yes | no | 1 or >1 | No | NR | 0.5 |
| US 4 Hemani, 1999 [34] | Undifferentiated conditions. | NP | yes | yes | no | >1 | Yes | PTCL/ GDL | 12 |
| UK 1 Campbell, 1998 [19-21,41, 43-45] | Coronary Heart Disease secondary prevention. | NP | no | no | yes | >1 | No | GDL | 12, 24, 56.4, 122.4 |
| US 3 Winter, 1981 [15] | Family planning, venereal diseases, acute non-traumatic minor illnesses. | RN | yes | no | no | 1 | NR | PTCL | 0-0.5 |
| US 2 Flynn, 1974 [35] | Undifferentiated. | RN | yes | no | yes | >1 | Yes | NR | ≥6 |
| US 1 Lewis, 1967 [23] | Hypertension, CVD, Obesity, Arthritis, Somatization. | LN | no | no | yes | >1 | No | GDL | 12 |

**Legend.**

US, United States; NL, The Netherlands; UK, United Kingdom; ZA, South Africa; RU, Russia; HIV, Human Immunodeficiency Virus; GORD, gastro-oesophageal reflux disease; COPD, Chronic Obstructive Pulmonary Disease; CVD, Cardiovascular Disease; Pulmonary Disease; BPLT, Blood Pressure Lowering Treatment; NP, nurse practitioner; NP+, nurse practitioner with higher degree/course; RN, registered nurse; LN, licensed nurse; 1^st^C, 1^st^ contact; UV, urgent visits; OC, on-going care; C (n), number of consultations; FCA, full clinical autonomy; GDLs, interventions based on clinical guidelines or protocols (PTL); FUP, follow-up episodes.

**Additional file, Table S6:** Sensitivity analyses.

| **Outcome** | **RCTs, n** | **SMD(95%CI)** | **Nurse patients, N** | **Physicians patients, N** | **Test for overall effect, Z** | **Heterogeneity:** |
| --- | --- | --- | --- | --- | --- | --- |
| **Satisfaction** | | | | | | |
| Original | 7 | 0.18 (0.13 to 0.23) | 2964 | 2857 | 6.70  (p< 0.00001) | Chi² = 65.97, df = 6  (P < 0.00001); I² = 91% |
| Sensitivity analyses | | | | | | |
| Excluding FCA or CANR | 6^†^ | 0.17 (0.12 to 0.22) | 2939 | 2832 | 6.34  (p < 0.00001) | Chi² =49.11, df = 5  (P < 0.00001); I² = 90% |
| Excluding quasi/cluster RCTs | 6^‡^ | 0.17 (0.12 to 0.23) | 2929 | 2847 | 6.50  (p < 0.00001) | Chi² = 59.06, df = 5  (P < 0.00001); I² = 92% |
| **Hospital Admissions** | | | | | | |
| Original | 5 | 0.76 (0.64 to 0.91) | 194/2108 | 210/1782 | 3.01  (p = 0.003) | Chi² = 4.30, df = 4  (P = 0.37); I² = 7% |
| Sensitivity analyses | | | | | | |
| Excluding FCA or CANR | 4^§^ | 0.75 (0.62 to 0.89) | 184/2068 | 208/1762 | 3.20  (p = 0.001) | Chi² = 1.56, df = 3  (P = 0.67); I² = 0% |
| **Mortality** | | | | | | |
| Original | 10 | 0.89 (0.84 to 0.96) | 1456/8094 | 1207/6558 | 3.27  (p = 0.001) | Chi² = 7.52, df = 9  (P = 0.58); I² = 0% |
| Sensitivity analyses | | | | | | |
| Excluding FCA or CANR | 8^║^ | 0.90 (0.84 to 0.96) | 1452/7630 | 1191/5871 | 3.12  (p = 0.002) | Chi² = 4.95, df = 7  (P = 0.67); I² = 0% |
| Excluding quasi/cluster RCTs | 8^¶^ | 0.85 (0.76 to 0.95) | 456/3001 | 2851 | 2.88 (p = 0.004) | Chi² = 6.81, df = 7  (P = 0.45); I² = 0% |
| Excluding potential cross-over^*^ | 8^#^ | 0.91 (0.85 to 0.97) | 1353/7271 | 1070/5588 | 2.72 (p = 0.006) | Chi² = 5.72, df = 7  (P = 0.57); I² = 0% |

**Legend.**

FCA, full clinical autonomy; CANR, clinical autonomy not reported; Chi², statistical test for heterogeneity; df, degrees of freedom; p, overall p-value for significance of effects of interventions; P, p-value of Chi² (evidence of heterogeneity of intervention effects); I², amount of heterogeneity between trials.

^*^Patients/clinicians crossover between groups.

^†^Without Winter, 1981 [15].

^‡^Without Du Moulin, 2007 [37].

^§^Without Flynn, 1974 [35].

^║^Without Voogdt-Pruis, 2010 [16] and Hemani, 1999 [34].

^¶^Without Fairall, 2012 [36] and Hemani, 1999 [34].

^#^ Without Hemani, 1999 [34] and Campbell, 1998 [19-21,41,43-45].

**Additional file, Table S7:** Individual trial estimates from data not combined in meta-analyses.

| **Study, year** | **Outcome** | **FUP, months** | **Nurse group** | **Physician group** | **OR/SMD/RR/Rate**  **(95% CI)** | **P** |
| --- | --- | --- | --- | --- | --- | --- |
|  |  |  | **n/N or mean (SD)** | **n/N or mean (SD)** |  |  |
| ***Hospital admissions*** |  |  |  |  |  |  |
| US 1  Mundinger, 2000 [22,24] | subsample (37%) of original patients who returned to the same clinician during phase two of trial and had hospitalisations | 24 | 10/222 | 15/184 | 0.55 (0.25 to 1.20) | 0.13 |
| US 1  Mundinger, 2000 [22,24] | subsample (37%) of original patients who returned to the same clinician during phase two of trial and had hospitalisations | 24 | 10/222 | 14/184 | 0.59 (0.27 to 1.30) | 0.19 |
| UK 1  Campbell, 1998 [19-21,41, 43-45] | all-cause hospital admissions | 120 | n=7647 | n=8642 | nr | 0.998 |
| US 1  Lewis, 1967 [23] | hospital admission rates per 1000 patient days | 12 | N=33 | N=33 | 34.3 vs 126.1 (CIs, nr) | NR |
| ZA 2  Fairall, 2012 [36] | incidence rate ratio of hospital admissions | 18 | 0.08(0.37), N=2582 | 0.09(0.43), N=2656 | 0.86 (0.80 to 0.96) | 0.005^*^ |
| ZA 1  Fairall, 2012 [36] | incidence rate ratio of hospital admissions | 16.3 | 0.19(0.54), N=4943 | 0.19(0.53), N=3407 | 0.91 (0.64 to 1.30) | 0.615^*^ |
| ***Mortality*** |  |  |  |  |  |  |
| UK 1  Campbell, 1998 [19-21,41, 43-45] | mortality due to coronary or non-fatal myocardial infarction | 56.4 | 100/673 | 125/670 | 0.80 (0.63 to 1.01) | 0.06 |
| UK 1  Campbell, 1998 [19-21,41, 43-45] | cumulative rate of all-cause mortality | 56.4 | 98/670 | 127/667 | 0.77 (0.60 to 0.98) | 0.03 |
| UK 1  Campbell, 1998 [19-21,41, 43-45] | cumulative rate of all-cause mortality | 120 | 254/673 | 277/670 | 0.91 (0.80 to 1.04) | 0.18 |
| UK 1  Campbell, 1998 [19-21,41, 43-45] | cumulative rate of mortality due to coronary or non-fatal myocardial infarction | 56.4 | 96/670 | 122/667 | 0.78 (0.61 to 1.00) | 0.05 |
| UK 1  Campbell, 1998 [19-21,41, 43-45] | cumulative rate of mortality due to coronary or non-fatal myocardial infarction | 120 | 196/673 | 195/670 | 1.00 (0.85 to 1.18) | 0.99 |

**Legend.**

US, United States; UK, United Kingdom; ZA, South Africa; FUP, follow-up; m, months; NLC, Nurse-Led Care; PLC, Physician-Led Care; N, total number of patients; n, number of patients or events; CI, confidence interval; df, degrees of freedom; OR, Odds Ratio; RR, Relative Risk; SMD, standard mean difference; SD, standard deviation; p, overall p-value; nr, not reported.

^*^Incidence rate ratio: Poisson regression models adjusted for intra-cluster correlation of outcomes, randomisation strata and for the duration enrolled in the trial.
